# Chromosome-scale assembly of the streamlined picoeukaryote Picochlorum sp. SENEW3 genome reveals Rabl-like chromatin structure and potential for C4 photosynthesis

**DOI:** 10.1099/mgen.0.001223

**Published:** 2024-04-16

**Authors:** Patrick A. da Roza, Héloïse Muller, Geraldine J. Sullivan, Roy S. K. Walker, Hugh D. Goold, Robert D. Willows, Brian Palenik, Ian T. Paulsen

**Affiliations:** 1 ARC Centre of Excellence in Synthetic Biology, Macquarie University, Sydney, NSW 2109, Australia; 2 School of Natural Sciences, Macquarie University, Sydney, Australia; 3 Institut Curie, PSL University, Sorbonne Université, CNRS, Nuclear Dynamics, 75005 Paris, France; 4 New South Wales Department of Primary Industries, Orange, NSW 2800, Australia; 5 Scripps Institution of Oceanography, University of California, San Diego, La Jolla, California 92093-0202, USA

**Keywords:** Photosynthetic picoeukaryote, genomic assembly, chromatin structure, C_4_photosynthesis, environmental adaption, *Picochlorum*

## Abstract

Genome sequencing and assembly of the photosynthetic picoeukaryotic *Picochlorum* sp. SENEW3 revealed a compact genome with a reduced gene set, few repetitive sequences, and an organized Rabl-like chromatin structure. Hi-C chromosome conformation capture revealed evidence of possible chromosomal translocations, as well as putative centromere locations. Maintenance of a relatively few selenoproteins, as compared to similarly sized marine picoprasinophytes Mamiellales, and broad halotolerance compared to others in Trebouxiophyceae, suggests evolutionary adaptation to variable salinity environments. Such adaptation may have driven size and genome minimization and have been enabled by the retention of a high number of membrane transporters. Identification of required pathway genes for both CAM and C_4_ photosynthetic carbon fixation, known to exist in the marine mamiellale pico-prasinophytes and seaweed *Ulva*, but few other chlorophyte species, further highlights the unique adaptations of this robust alga. This high-quality assembly provides a significant advance in the resources available for genomic investigations of this and other photosynthetic picoeukaryotes.

**Impact Statement**

This work provides a fully annotated chromosomal genome assembly of euryhaline pico-sized green algae. It identifies key genomic features, including putative centromeres and chromosomal translocations, reduced selenoprotein complement, CAM and C_4_ photosynthetic carbon fixation gene sets. This study adds to the field by identifying novel genomic aspects of this organism, affords additional utility in genomic studies via the genome’s complete functional annotation, and provides a basis for further investigation of algal genomic architecture.

## Introduction

The green algae are a highly successful group of photosynthetic eukaryotes within the green lineage (Viridiplantae). Comprising of two divergent clades, Chlorophyta and Streptophyta (which includes the charophyte algae and land plants); the green algae display a high level of phylogenetic, ecological, and morphological diversity [1,2]. Rapid advances in DNA sequencing over the past decade have facilitated the genomic sequencing and analysis of numerous green algal species, enabling investigation of many biological facets of these organisms, including ecological adaptations [3,4], photosynthetic evolution [5,6], cellular mechanisms and functions [7,8], evolutionary phylogeny [2,9], and the biosynthesis of valuable metabolites and compounds [4,10].

Photosynthetic picoeukaryotes (PPEs) are a widely distributed and metabolically diverse group of photosynthetic unicellular algae approximately 1–3 µM in diameter, that have independently evolved within heterokont, haptophyte and green algal lineages [11,12]. They are important primary producing organisms, forming key components of aquatic ecosystems and microbial food webs [13,16]. Their highly reduced size is posited as being a primary factor in their success and broad environmental distribution. Such miniaturization can reduce predation by some grazers, and results in a high cell surface area to volume ratio that enhances the efficiency of nutrient uptake and transport [11]. These attributes enable persistence in oligotrophic conditions and high-specific growth rates under nutrient-rich regimes [17,18].

*Picochlorum* is a genus of picoeukaryotic green algae in the class Trebouxiophyceae, that have recently been of interest in microalgal research [19,21]. Isolates of the genus, predominantly inhabit marine and brackish water habits, and display broad levels of halo- and thermotolerance. Similar to the smallest known PPEs, of the Mamiellophyceae genus *Ostreococcus* [22,25], *Picochlorum* are non-flagellated pico-sized unicells with a mean diameter of 2 µM; and possess correspondingly small genomes in the range of 13–15 Mbp [20,23, 24, 26,28]. However, unlike *Ostreococcus,* isolates of *Picochlorum* exhibit great flexibility in growth conditions, and rapid growth rates under high-light, and nutrient-rich conditions [29,30]. Due to their streamlined genomes, robust characteristics, and vigorous growth rates, *Picochlorum* isolates have recently been investigated for comparative genomics analysis [31], environmental fluctuations and toxicity [26,28, 32], photophysiology [33], biomass and lipid production [29,34,41], aquaculture feedstock, waste management and bioremediation [42,46], as well as biofuels, transgene expression [47,50] and most recently adaptive laboratory evolution (ALE) [51].

The isolate *Picochlorum* sp. SENEW3 (*P. SENEW3*) exhibits particularly robust characteristics under variable environmental conditions. It tolerates temperatures between 16–32 °C and thriving in both freshwater, three-fold seawater salinity under laboratory conditions, and seasonal salinity fluctuations of 0.2 to 13 Brix units in its natural estuary pond environment [27]. Previous examination of the *P. SENEW3* genome revealed its small and compact nature, enriched with putative salt stress response transporters, functional gene clusters and multiple instances of horizontal gene transfer related to stress adaptation [26,52]. As such, it provides an intriguing case of picoeukaryote genome reduction under challenging environmental conditions, with robust growth and stress responses that appear to be absent in other PPEs such as *Ostreococcus*.

Previous genomic investigations have been partially limited due to fragmented short-read assembly technologies or incomplete functional annotation. However, recent advances in the availability of long-read sequencing [53,54], chromosome conformation capture techniques [55,59], and eukaryotic genome annotation pipelines [60,63] now enable full chromosomal assembly and functional annotation of even large, repeat rich plant genomes, improving omics analysis of genomic features [57,64,66]. Utilizing both short-read and long-read Oxford Nanopore Technologies (ONT) DNA sequencing, pulse-field gel electrophoresis (PFGE), Hi-C chromatin conformation capture and functional annotation pipelines, this work presents in-depth investigation and functional analysis of the *P. SENEW3* genome with a fully annotated chromosomal assembly. This provides a key resource for further omics and molecular analysis of this robust poikilohaline picoeukaryote alga.

## Methods

### *Picochlorum* sp. SENEW3 cell culture

*Picochlorum* sp. SENEW3 (*P. SENEW3*) was obtained from Professor Brian Palenik at Scripps Institution of Oceanography, UC San Diego, CA, USA and was originally isolated from a poikilohaline pond in the San Elijo Lagoon estuary in southern California [27]. Cultures were grown in f/2 medium without the addition of sodium silicate [67,68] and with artificial seawater base used for Keller (K) medium [69]. Cultures were grown at 22 °C, 100 r.p.m., 100 m^−2^ s^−1^ white light on a 14 hour day: 10 h night light cycle and transferred weekly to fresh medium. Cultures were periodically aseptically cell sorted with a BD Influx flow cytometer and/or treated with antibiotics (50 µg ml^−1^ ampicillin, 10 µg ml^−1^ gentamicin, µg ml^−1^ kanamycin, and 100 µg ml^−1^ neomycin) [70] to prevent bacterial or fungal growth. Cell-density estimations via haemocytometer cell count were made using an Olympus BH-2 microscope with a ‘Neubauer improved bright-line’ haemocytometer slide as per the manufacturer instructions.

### Pulsed-field gel electrophoresis

Genomic DNA agarose plugs were generated based on the protocol of Hage and Houseley [71]. Briefly, *P. SENEW3* cells were cultured in f/2 medium to mid exponential growth phase cell density of 2×10^7^ cells ml^−1^. Ten ml cell-culture aliquots were centrifuged at 3200 ***g*** for 15 mins, cell pellets were washed in 1 ml of wash buffer (10 mM Tris pH 7.6, 50 mM ethylenediaminetetraacetic acid [EDTA]) and resuspended in 50 µl of wash buffer with 1 mg ml^−1^ lyticase. Cells were heated to 55 °C for 5 mins and mixed with 55 µl of melted 1.6 % SeaKem LE agarose in dH_2_O at 55 °C and set in PFGE combe wells at 4 °C for 1 h. Solid plugs were transferred to 1.5 ml microcentrifuge tubes, digested in 500 µl wash buffer with 1 mg ml^−1^ lyticase for 3 h at 37 °C and then in 500 µl PK buffer (100 mM EDTA, 0.2 % sodium deoxycholate, 1 % sodium lauryl sarcosine) with 1 mg ml^−1^ proteinase K (Sigma-Aldrich P6556) overnight at 55 °C. Digested cell plugs were washed three times in 1 ml wash buffer for 30 mins at room temperature, resuspended in 500 µl wash buffer and stored at 4 °C.

*P. SENEW3* chromosomal DNA was separated using a Bio-Rad CHEF Mapper XA PFGE system. Four agarose plugs and a NEB Yeast Chromosome PFGE marker (N0345S) plug were inserted into a 1 % SeaPlaque Low Melting Point Agarose (Lonza: 50101–25 g) gel, prepared with 0.5 X Tris/Borate/EDTA (TBE) buffer. Each plug was run in parallel for 24, 28, 32 and 36 h, respectively (Table S1, available in the online version of this article). Following each time point, each lane was excised and visualized with a blue light gel box.

### Genomic DNA extraction

Genomic DNA for ONT long-read sequencing was extracted via agarose gel electrophoresis concentration and drop dialysis of PFGE agarose plugs as described in Sambrook and Russell [72] protocol 20 [72]. Ten *P. SENEW3* PFGE plugs were placed together within a large cut-out well in 5 % NuSieve GTG Agarose gel (Lonza 50081), stained with SYBR Safe DNA gel stain (Invitrogen S33102) and separated using standard agarose gel electrophoresis apparatus at 60 V for 3 h at 4 °C. The resulting gel band was visualized using a blue-light gel box, excised, and stored with 1 ml of PFGE wash buffer at 4 °C. Gel slices were equilibrated in 12 ml of equilibration buffer (1X TBE, 100 mM NaCl, 30 µM spermine, 70 µM spermidine) for 20 mins at room temperature with gentle agitation, and then melted at 68 °C for 15 mins followed by incubation for 2 h at 42 °C with 10 units of β-agarase I (New England BioLabs M0392S). Digested supernatant (100 µl at a time) was spotted onto the centre of a 0.05 µM MF-Millipore membrane filter (Merck Millipore VMWP02500) floating on 100 ml of transfection buffer (10 mM Tris pH 7.5, 0.1 mM EDTA, 100 mM NaCl, 30 µM spermine, 70 µM spermidine), dialysed for 1 h at room temperature, followed by replacement with the same volume of fresh buffer and an additional 1 h of dialysis. DNA was transferred into a new 2 ml Eppendorf tube and precipitated with 3 volumes of 100 % cold ethanol and 1/10 vol 3 M Sodium Acetate for 1 h at −30 °C. DNA precipitate was pelleted at 12 000 ***g*** for 15 mins, washed three times with 70 % ethanol and resuspended in 100 µl Tris-EDTA buffer overnight at 4 °C. DNA quality and concentration were assessed via Nanodrop and Qbit dsDNA HS Assay (Invitrogen, Thermo Fisher Scientific: Q32854). Genomic DNA integrity was assessed via agarose gel electrophoresis.

### Genomic sequencing

Genomic DNA for Illumina DNA sequencing (used for genome polishing and organelle assembly) was extracted via a CTAB/phenol/chloroform method based off Jagielski *et al*. [73]. In short, 100 ml of approximately 1×10^7^ cell ml^−1^ of *P. SENEW3* were centrifuged at 3200 ***g*** at 4 °C for 15 mins, resuspended in 600 µl of freshly prepared DNA extraction buffer (10 mM Tris-HCL pH 8.0, 1 mM EDTA, 100 mM NaCl, 2 % Triton-x100, 1 % SDS) and transferred to 2 ml Eppendorf tubes with 0.1 mm glass beads (cell pellet/beads, 1 : 1 ratio). Sample tubes were vortexed at 20 Hz for 5 mins, lysate was transferred to 5 ml Eppendorf tubes, and remaining glass beads were washed twice with 250 µl of extraction buffer that was pooled with the initial lysate. Samples were incubated at 56 °C for 1 h with 160 µg ml^−1^ proteinase K, followed by the addition of 2 µl of 2-mercaptoethanol, 500 µl of 5 M NaCl, 400 µl of prewarmed 10 % CTAB [10 % Cetyltrimethyl ammonium bromide and 0.7 % NaCl (w/v) in nuclease free water] and incubated at 65 °C for 10 mins. Lysate was extracted once with chloroform: isoamyl alcohol (24 : 1 v/v), mixed via inversion and centrifugation at 12 000 ***g*** for 5 mins, supernatant was aspirated and extracted three times with equal volume of phenol: chloroform: isoamyl alcohol (25 : 24 : 1 v/v/v). Genomic DNA was precipitated with three volumes of 100 % ethanol and 1/10 vol 3 M Sodium Acetate for 1 h at −30 °C. DNA precipitate was pelleted at 12 000 ***g*** for 15 mins, washed three times with 70 % ethanol and resuspended in 100 µl Tris-EDTA buffer overnight at 4 °C. Residual RNA was degraded with ten units of RNase I (Thermo Scientific EN0601) at 37 °C for 1 h followed by ethanol precipitation and wash was repeated, and DNA was resuspended in 100 µl Tris-EDTA buffer. DNA quality and concentration was assessed via Nanodrop and Qubit dsDNA HS Assay (Invitrogen, Thermo Fisher Scientific: Q32854). Genomic DNA integrity was assessed via agarose gel electrophoresis. DNA library was prepared using TruSeq Nano DNA Kit (preparation guide part no. 15 041 110 Rev. D) and Illumina pair-end DNA sequencing was undertaken by Macrogen (Republic of Korea).

Long-read sequencing of genomic DNA was undertaken using ONT MinION for nuclear genome assembly. Then, 1 µg of gDNA was sequenced as per ONT protocol ‘1D Genomic DNA by ligation’ (SQK-LSK109) version: GDE_9063_v109_revD. In short, gDNA was repaired and end-prepared using NEBNext FFPDNA Repair Mix and NEBNext End repair/dA-tailing Module in accordance with the manufacturer’s instructions and size selected with 1×volume (1 : 1/bead : sample ratio, 60 µl total) of AMPure XP magnetic beads. Adapter ligation and clean-up was carried out with NEBNext Quick Ligation Module (E6056) T4 Ligase and purified with 0.67 X volume (beads:sample, 40 µl) of AMPure XP magnetic beads. DNA was then prepared for sequencing using the provided ligation sequencing kit (SQK-LSK109), primed, and loaded on to the SpotON flow cell (FLO-MIN106, v. R9.4.1). MinION sequencing was run with the MinKnow software v 3.3.2 for 48 h (base voltage −180 mV, 1.5 h mux scan time). Raw data was output as fast5 files and converted to fastq via MinKnow software base-calling (guppy base-caller v. 3.0.3).

### Genome assembly

Organellar genomes were constructed by mapping assembly of the Illumina pair-end reads. Here, Mirabait (MIRA v 4.9) [74] was used to pull out Illumina reads that mapped to the chloroplast and mitochondria of the first assembly of the *P. SENEW3* genome [26]. MIRA v 4.9 was then used to re-assemble the baited reads into chloroplast and mitochondria genomes.

The *P. SENEW3* nuclear genome was assembled *de novo* with the ONT MinION sequencing reads and was polished with both the ONT and the Illumina sequencing reads. ONT reads were assembled with Canu v 1.8 using default settings and corrected long-reads were remapped to the assembled contigs with Minimap2 v 2.17 [75], which were then polished with Racon v 1.4.3 [76]. Racon polished contigs were then polished once with Medaka v 1.0.3 [77]. The resulting contigs were iteratively corrected four times with indexed Illumina short-reads (Samtools v 1.9 [78,79]) using Pilon v 1.23 [80]. The resulting alignments were inspected and manually corrected with Gap5 (Staden Package v 1.9) [81]. Contigs with unmapped or poor read coverage were discarded as likely mis-assemblies or contaminants.

### Hi-C: chromosome conformation capture

In total, 150 ml of approx. 1×10^7^ cell ml^−1^ *P*. *SENEW3* culture in f/2 medium were fixed directly in culture medium by adding methanol stabilised formaldehyde (Sigma-Aldrich F8775−4×25 ml) to each cell culture to a final concentration of 3 % (v/v). Cultures were incubated for 30 mins at room temperature (RT), and subsequently quenched with 2 volumes of 2.5 M glycine for 5 mins at RT then for 15 mins at 4 °C. Cells were centrifuged at 3200 ***g*** for 15 mins at 4 °C, washed with 20 ml of f/2 medium, re-centrifuged at 3200 ***g*** for 5 mins at 4 °C. Samples were then resuspended in 1 ml of f/2 medium, transferred to 1.5 ml Eppendorf tubes, centrifuged for 2 mins at 13 000 ***g***, supernatant was aspirated and pellets were frozen and stored at –80 °C. Samples were shipped on dry-ice to the Curie Institute, Centre of Research – UMR3664 Nuclear Dynamics – Paris France for Hi-C chromosome conformation capture processing.

The cell pellet was thawed on ice and suspended in 500 µl TE +cOmplete, EDTA-free Protease Inhibitor Cocktail (Roche). Sample was then treated for three cycles of 20 s at 6.5 M/s with 0.5 mm beads in a FastPrep apparatus (MP Biomedicals) to break the cell walls before starting the Hi-C protocol. Hi-C was carried out using the Arima-HiC +kit (Arima Genomics) following the manufacturer’s instructions. The Hi-C library was processed according to Arima Genomics recommendation using the KAPA HyperPrep Kit (Roche) and Illumina TruSeq sequencing adapters (Illumina 20020590) to make an Illumina sequencing library that was further sequenced as PE100 on a NovaSeq 6000 system at the Curie CoreTech platform.

Hi-C pair-end reads were aligned and filtered using HiC-Pro [82]. Valid pairs were then processed to build raw and balanced Hi-C matrices in .cool format using the cooler package [83]. Hi-C matrices were further manipulated and drawn at indicated resolutions using cooler, cooltools (version 0.5.0, doi:10.5281/zenodo.5708875) and matplotlib (version 3.3.2, doi:10.5281/zenodo.5773480) [84] python packages and library.

### Genome annotation

*P. SENEW3* genome annotation was undertaken separately for organellar and nuclear genomes. The chloroplast and mitochondrial assemblies were annotated with Prokka: rapid prokaryotic genome annotation v 1.14.6 [85]. Annotation of the nuclear genome was made using the ‘assemblage’ iterative two-pass workflow (https://github.com/sujaikumar/assemblage) of the MAKER2 eukaryotic genome annotation pipeline [63]. Prior to running the first pass of the MAKER2 v 3.01.03 genome annotation pipeline, separate eukaryotic *ab initio* gene predictions were run, and a custom repetitive sequence library was generated for the *P. SENEW3* nuclear genome. Augustus v 3.3.3 gene prediction [86] was trained on the first version of the *P. SENEW3* genome (psev1) [26] with the psev1 protein and transcript/ESTs as evidence for prediction training. Scipio v 1.4 [87] was used to map protein and transcript evidence to the psev1 genome to generate a gene training set. Augustus v 3.3.3 e-training and optimization scripts were used to generate an Augustus species gene prediction parameter set. BUSCO v 3.0.2 was used to generate the in initial *ab initio* predicted gene set for the *P. SENEW3* genome, using the eukaryote core BUSCO dataset (v eukaryote_odb9 2016-11-02) [88] and the Augustus v 3.3.3 generated psev1 species parameters set. BUSCO gene predictions were converted from gff3 file type to SNAP v 2006-07-28 [89] gene predictor compatible hmm file type with an in-house Perl script. GeneMark-ES v 3.60 [90] with default setting was run against the *P*. SENEW3 nuclear genome as the third *ab initio* gene predictor. A custom repetitive sequence library was generated for the *P. SENEW3* nuclear genome using the MAKER2 ‘repeat library construction-advanced’ protocol [91].

BUSCO-SNAP predictions, GeneMark-ES predictions, the custom repeat masking library, psev1 EST’s and protein data sets from *Picochlorum* sp. SENEW3 v 1.0 [26], *Picochlorum* sp. SENEW3 v 2.0, *Picochlorum* nbrc102739, *Picochlorum oculatum*, *Picochlorum oklahomensis* [52], *Picochlorum soloecismus* (spDOE101) [92] and *Picochlorum renovo* v 2 [47] were imported into the first pass of the MAKER2 v 3.01.03 pipeline. MAKER2 first-pass output was used to re-train SNAP gene predictor. MAKER2 pipeline was run for an increased stringency second pass with updated SNAP, Genemark and Augustus. Putative functions and domain information were appended to MAKER2 gene predictions using BlastP (blast suite v 2.10.1+) [93] against the UniProt/SwisProt database [94] and InterProScan v 5.42.78.0 [95]. Genomic tRNAs were annotated with tRNAscan-SE v 2.0 [96].

### Genomic analysis

The combined nuclear, chloroplast and mitochondrial genome assemblies were assessed for assembly completeness using BUSCO v 3.0.2 [97] against the eukaryota_odb9 (2016-11-02) BUSCO gene database. RepeatModeler2 v 2.0.1 [98] was run against the combined genome to produce a custom *de novo* transposable element (TE) family library. The TE library was input into RepeatMasker v 4.1.0 (database: Dfam_3.1, rmblastn version 2.10.0+) [99], which was used to assess the type and coverage of repetitive elements present in the assembled genome (Table S4). Comparative gene orthology across 15 select species/strains of the phylum Chlorophyta (Table S19) with available proteomes was analysed using Orthofinder v 2.5.4 [100,101]. Carbohydrate-active enzymes were identified via dbCAN2 meta server via protein sequence with HMMER (E-Value <1e-15, coverage >0.35), DIAMOND (E-Value <1e-102), hotpep (Frequency >2.6, Hits >6) and CGCFinder (Distance<=2, signature genes=CAZyme+ TC) [102,103] (Table S12). Transporters were identified using TransAAP v. 2.0 [104]. Variant calls, including identification of SNPs, insertion and deletions (INDELs) were made using bcftools v. 1.17 (Tables S21 and S22) [79]. Ploidy analysis was undertaken using nQuire [105], kmc tools and smudgeplot [106] and manual examination of variant calls. Additional functional annotations of predicted proteins were identified using KEGG GhostKOALA [107], and eggnog-mapper v. 2.1.6 [108,109] (Tables S9–11). A list of combined annotation available in Table S3.

## Results and discussion

### Gene content

**Fig. 1.**
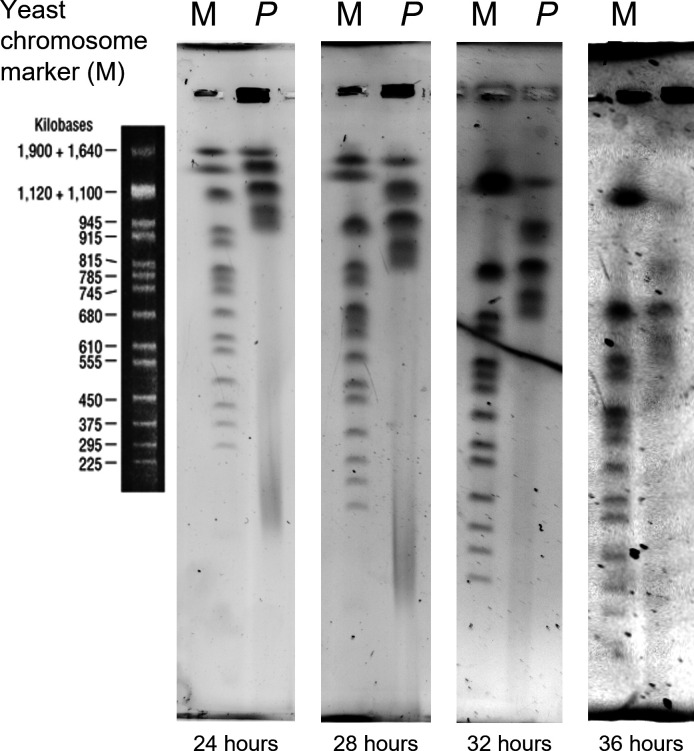
Pulsed-field gel electrophoresis gel slices of *P. SENEW3* chromosomal DNA (**p**) against NEB Yeast Chromosome PFGE marker N0345S (**m**) at 24, 28, 32 and 36 h time points, using a Bio-Rad CHEF Mapper XA pulsed-field gel electrophoresis system.

Using a combination of short reads (Illumina), long reads (ONT MinION) and proximity information provided by a Hi-C experiment (see paragraph below), we assembled the 13.75 Mbp primary haploid assembly of the *P*. SENEW3 nuclear genome into 12 chromosomes (chr) ranging from 0.77 to 1.58 Mbp. These were consistent with the range of physical chromosomes separated by PFGE (Fig. 1). Variant calling to determine heterozygosity, identified 964 SNPs (7.02 per 100 Kbp) and 366 INDELs (2.66 per 100 Kbp) across the primary assembly (summarized in Table S2). This rate, for example is higher than that of similarly sized *Saccharomyces cerevisiae* genome of four SNPs per 100 Kbp [110]. Bioinformatic ploidy analysis (Table S20) and examination of variant calls indicated the *P. SENEW3* genome is at least diploid. Manual examination of the variants did reveal some loci to have SNPs occurring in approximate 25 % : 75 % ratios. This could be indicative of tetraploidy, however distinguishing between diploids and tetraploids can be difficult and this seems the less likely scenario based on the current and previous analysis [52]. It is notable that previous examination of heterozygosity among *Picochlorum* strains has identified significant variation in genome size, ploidy, and heterozygosity across isolates. Among the putative diploids it appears that the genome of *P. SENEW* possesses relatively low heterozygosity [52]. The nuclear genome had an average G+C content of 46.3 % and BUSCO (Benchmarking Universal Single-Copy Orthologs) completeness score of 90.7 % (C:90.7 % [S:90.4 %,D:0.3 %], F:1.0 %, M:8.3 %, n:303) [88]. Organellar 74.135 Kbp chloroplast and 37.566 Kbp mitochondria genomes had G+C contents of 32.2 and 42.4 %, respectively (Table S2).

**Fig. 2.**
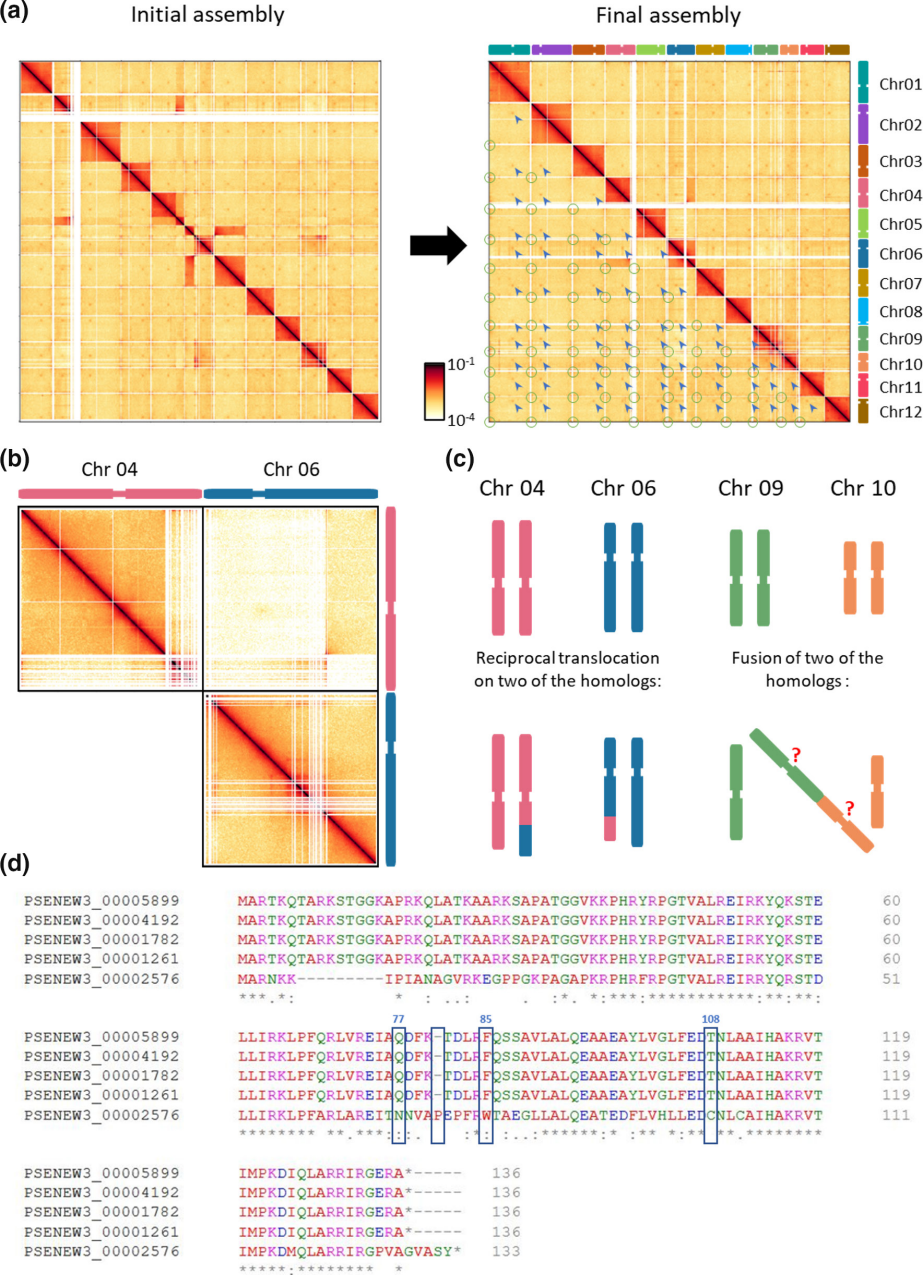
(**a**) Hi-C contact heat map at 20 Kbp resolution displaying initial and final nuclear genome primary assembly (Ch01–Chr12) with putative centromeric regions (blue arrows) and telomeric contacts (green circles). Scale bar displays frequency of contact counts (log scale) per 5 Kbp sequence bin. (**b**) Hi-C contact heat map at 5 Kbp resolution of Chr04, Chr06 and Chr04-Chr06 interaction. (**c**) Representation of possible reciprocal translocation between two homologues of Chr04 and Chr06, and chromosomal fusion of two homologues between Chr09 and Chr10. (**d**) Protein sequence alignment (Clustal Omega v 1.2.4) of *P. SENEW3* histone H3 homologues (PSENEW3_00005899, _00004192, _00001782, _00001261) and putative CenH3/CENP-A histone H3 variant (PSENEW3_00002576), highlighting key CenH3 amino acid substitutions (sites 77, 85 and 108) and insertion [138].

**Fig. 3.**
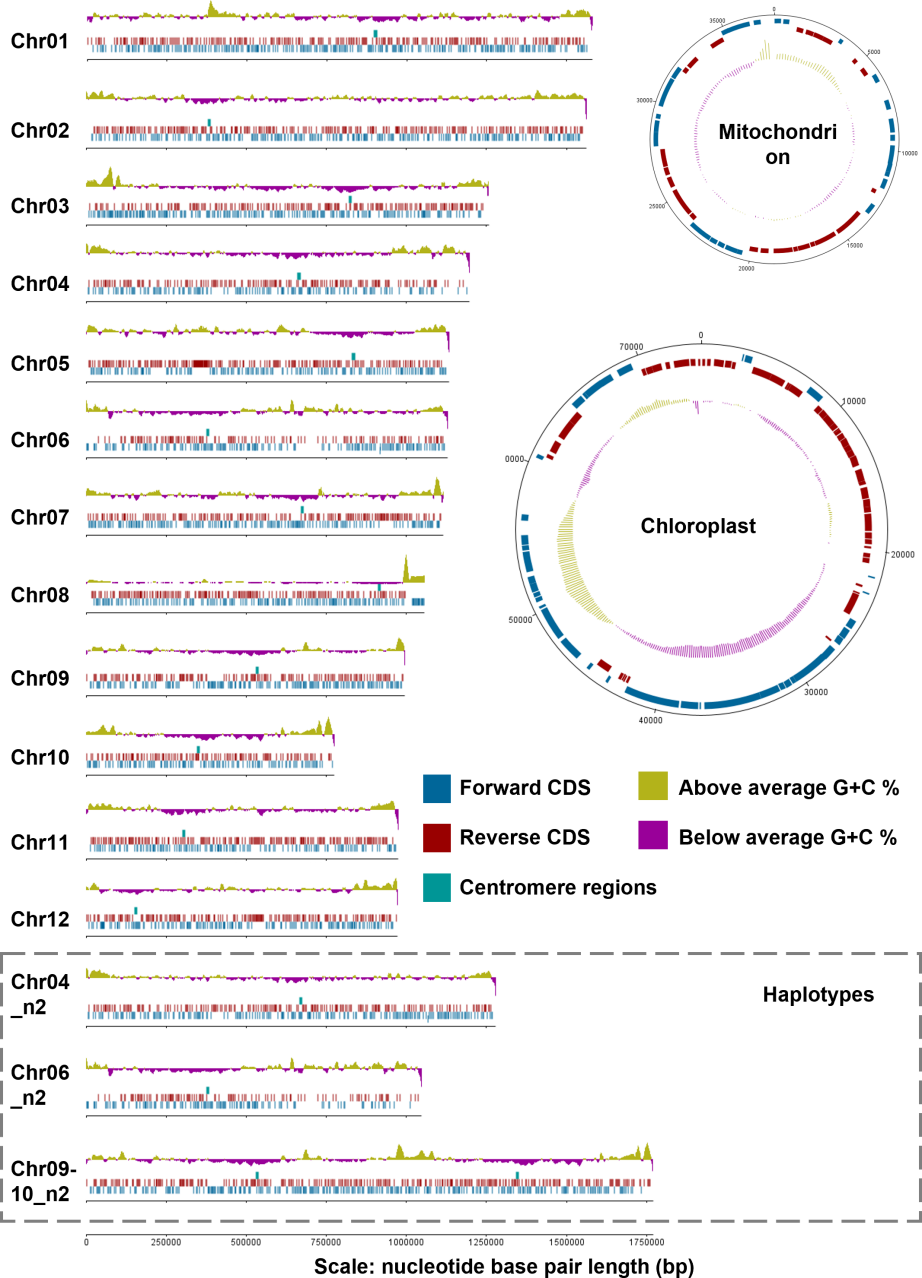
Genome chromosome and organelle map, displaying chromosomes (Chr) 1–12, chromosome haplotypes (**n2**), chloroplast and mitochondria sequence lengths, created with Artemis - DNA plotter [208]. Forward protein coding sequences (CDS) shown in blue, reverse CDS in red, putative centromere regions in green, above average G+C % in yellow, and below average G+C % in purple. The Chr09-10_n2 haplotype represents a putative fusion. Gene loci for primary and haplotype assemblies available in Tables S7 and S8.

Repetitive sequence analysis of the assembled genome using Repeatmasker – based off the generated Repeatmodeler2 TE library, revealed a low density of repeat elements. TEs made up only 4.99 % of the assembled nuclear genome (5.23 % including organelle genomes) and were comprised primarily of unclassified repeats (1.87 %) and retroelements (2.06 %), including long interspersed repetitive elements (1.25 %, LINEs) and long terminal repeats (0.81 %, LTRs). DNA transposons only made up 0.35 % of the nuclear genome (Table S4). Despite this low TE count, several localized genomic regions appear to be enriched in repetitive sequences, likely the cause of difficulty in assembly and Hi-C contact mapping. Three such regions included the right arms of chr04 and ch06 resulting in fragmented Hi-C contact mapping (Fig. 2b), and a ~10 Kbp region of Guanine (G) repeats resulting in a high G+C % spike at the right arm of chr08 (Fig. 3). Thus, the *P. SENEW3* assembled genome is in general repeat poor, however it is likely that the repeat count is underrepresented. Nevertheless, the *P. SENEW3* genome appears to be highly compact like that of other PPE’s (e.g. *O. tauri* and *Micromonas*) [9,23]. In contrast to many other green lineage organisms, which typically contain many repeat sequences and transposable elements, the number of which is known to be positively correlated with genome size above ~10 Mbp [111,112].

**Fig. 4.**
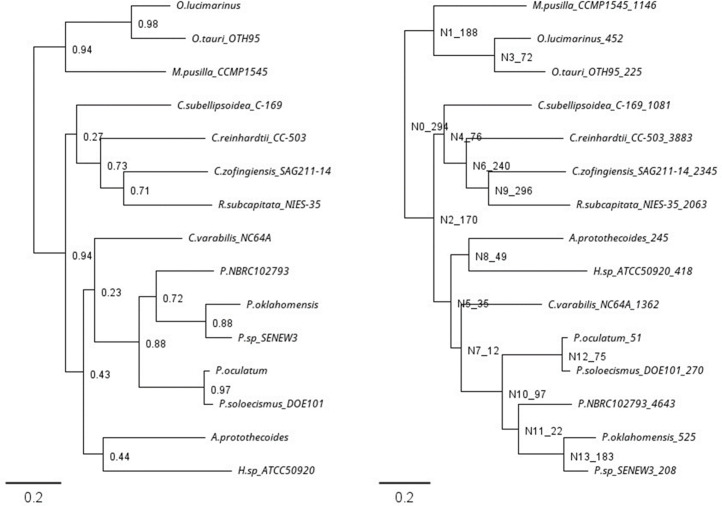
Orthogroup analysis of 15 representative chlorophyte proteomes displaying (a) an inferred species tree based on orthogroup relationships and (b) the number of gene duplications with greater than 0.5 support per orthogroup and species. Scale bar displays the number of amino acid substitutions per site.

A total of 6564 genes were predicted across the nuclear and organellar genomes. This represents fewer predicted genes than earlier assemblies [26,52], however increased prediction stringency applied during the second pass of the MAKER2 workflow successfully removed false-positive gene predictions (923) from the first run of the annotation pipeline. Final density represents one gene per 2,161 bp, with 74.35 % of the genome represented by protein-coding sequence. This high gene-density is comparable to that of the Mamiellophyceae genomes of *Ostreococcus* and *Micromonas* with protein-coding sequence comprising close to 70 % for most strains [1,9, 22,24]. Whole proteome orthogroup analysis against 14 other Chlorophyta species with complete genome assemblies was used verify the phylogeny of the assembly. This clearly placed the *P. SENEW3* within the *Picochlorum* genus and most similar to *Picochlorum oklahomensis* (Fig. 4a). In addition, the relatively low number of gene duplication events in the *P. SENEW3* genome (Fig. 4b) was consistent with its streamlined and compact nature. Meiosis is a key process in evolutionary diversification in eukaryotic organisms, however in several groups of green algae, sexual reproduction is either absent or yet undocumented, as appears to be the case for most planktonic species [1,113]. The presence of core meiotic determinant genes (though many more are involved in the process) [114,115] was examined to assess whether *P. SENEW3* is likely to undertake sexual reproduction. Several of these were identified: *DMC1*, *HOP2*, *MSH5* (and homologues *MSH2* and *MSH6*) *SPO11-2*, *HAP2* and *MER3*; yet six others were missing (Table S13), suggesting but leaving uncertain whether *P. SENEW3* can undergo meiosis.

The occurrence of such highly reduced genome and cell size has led to the proposition that picoeukaryotic algae may be near the limit for minimal genomes of free-living photosynthetic eukaryotes [1]. One hypothesis explaining this genome reduction in PPEs is the Black Queen hypothesis (BQH), which predicts that an availability of shared metabolites in marine environments would drive the loss of redundant metabolic pathways in individual genomes/species that are otherwise available within the surrounding microbial community. Providing a selective advantage by conserving an organism’s finite resources through adaptive gene loss [116,118]. However, unlike other PPEs, such as *Ostreococcus*, *P. SENEW3* displays robust growth in variable environmental conditions, tolerating both euryhaline conditions and temperatures between 16–32 °C [26]. In such extreme or challenging environments, this pressure for adaptive gene loss should be reduced since there are typically fewer community members to produced expensive metabolites [116,118]. Hence, the large range of environmental tolerances of *P. SENEW3* and payoffs between growth, nutrient availability, and stress, presents an alternate environmental adaptation paradigm for PPEs.

### Genome assembly and centromere identification with Hi-C

Chromosome conformation capture (3C) techniques such as Hi-C allow for the examination of spatial chromatin organization by quantifying the interactions between genomic loci that may otherwise be distantly separated in sequence location [55,56, 119, 120]. This is based on the principle that interaction frequencies decrease with genomic distance and that *cis*-interactions (from loci located on the same chromosome) are always significantly higher than *trans*-interactions (from loci located on different chromosomes). Recently developed Hi-C based genome assembly programmes [121,123] have proved to be a powerful instrument in the genomics toolkit, and are being rapidly integrated into individual organism sequencing projects [57,65, 66, 124, 125] and large-scale biodiversity sequencing projects such as the earth biogenome project [126,128]. In particular, Hi-C assembly allows for chromosome scaffolding without the need of contiguous sequences and gap filling. This alleviates difficulties related to large repetitive sequences that often stay unassembled in genomes.

For the small, repeat-poor *P. SENEW3* genome, we corrected minimal scaffolding errors via visual inspection of the Hi-C matrix mapped to our initial reference assembly (Fig. 2a). This resulted in a final reference genome with 12 chromosomes, corresponding to 12 individual squares of higher interaction frequencies along the diagonal of the matrix (Figs 2a and S1). Still, an abnormal signal is present in the inter-chromosomal space between chr04 and chr06 highlighted on a higher resolution map (5 kbp bins) in Fig. 2b. The end of the right arm of chr06 (~327 Kbp) makes high interactions with the rest of chr06, but also with a large segment of chr04. Similarly, the end of the right arm of chr04 (~245 Kbp) makes high contacts with chr06. Nevertheless, these two regions do not interact together more than in the inter-chromosomal range. This represents a reciprocal translocation between a subset of chr04 and chr06 in the population. Either this translocation occurred early during the growth of the clone used to perform the Hi-C experiment and represent a large part of the final analysed population, or the strain is diploid, and the reciprocal translocation occurred between two of the homologs (Fig. 2c). Another signal appears to be unusual in the inter-chromosomal area on the final assembly, between chr09 and chr10. The contact frequency between the two chromosomes is in between the inter-chromosomal range of frequencies with other chromosomes and the intra-chromosomal range. We explain this signal by a fusion occurring between both chromosomes but involving only part of the population, or again, the fusion of two homologues only in a diploid strain (Fig. 2). Additional Hi-C experiments with a fresh clone, could be used to decipher the validity of these hypotheses and whether the genome is inherently unstable under laboratory culture, or such rearrangements are fixed within the population. We have not undertaken the work to address these hypotheses further, favouring the rearrangement of homologues in a diploid strain (Fig. 2c). This is supported by the existence of a PFGE chromosomal band above 1640 Kbp (Fig. 1), which would correspond to the ~1770 Kbp sequence length of chr09-10_n2 (Fig. 3). We have therefore provided alternative sequences and matrix for chr04, 06, 09 and 10 that we named with the suffix ‘n2’ (Fig. S2).

One of the most striking architectural chromosomal features visible on Hi-C matrices are inter-chromosomal centromere interactions. These interactions are the consequence of centromere clustering, and a broadly shared organization across the eukaryotic domain of life [129]. Several groups have taken advantage of this property to identify centromeric regions in species where they were not defined [130,134]. In *P. SENEW*, the inter-chromosomal area clearly shows such a dotty pattern between chromosomes (blue arrowheads on Fig. 2a). This enabled confirmation of 12 centromeric regions (primary assembly), one per chromosome, marked by high-frequency trans-interactions. Sequence bins of high-interaction chromosomal *trans*-signals were identified using matrices at 10 kb resolution and defined as putative centromeric regions (Table S5).

To further characterise the centromeres of *P. SENEW3*, and decipher whether they exist as point centromeres, as in budding yeasts, or short regional centromeres (below 10 kb) [129,135] additional experiments are required. Chromatin immunoprecipitation using a tagged version of the centromeric variant of the histone H3 (CENH3/CENP-A) as a bait has been successfully used for this purpose [136,137]. To initiate this future work, the genome was examined for a putative CENH3. A tblastn homology search using human H3.1 (UniProt P68431 H31_HUMAN) as query against the *P. SENEW* genome identified five H3 homologues (Fig. 2d, and Table S6). Four of the identified proteins were identical to each other and contained only four mismatches compared to human H3.1: PSENEW3_00005899, PSENEW3_00004192, PSENEW3_00001782, PSENEW3_00001261. Corresponding genes were already well annotated in our database as being H3 homologues, belonging to the four histone clusters of the genome (each comprising H3, H4, H2A and H2B). The last homologue, PSENEW3_00002576, had diverged amino acid sequence, was found isolated on chr01, and had two introns while the others had none. Additionally, multiple alignment of this proteins revealed substitutions in PSENEW3_00002576 (blue boxes on Fig. 2d) at key positions reported to be mutated in CENH3s compared canonical H3s in other species [[138]], as well as a higher divergence in the N-terminal tail. Taken together, these characteristics gave sufficient evidence to annotate PSENEW3_00002576 as a putative CENH3 in *P. SENEW3*.

Hi-C analysis of additional architectural features at 5 kb resolution indicated that the *P. SENEW3* genome possessed few other elements of chromosome organization commonly found in other species such as chromatin compartmentalization or topologically associated domains (TADs) [120,139, 140]. Nevertheless, two other feature types were identified. The first of these were several clear loop anchors, the most prominent involving three regions on chr12 (Fig. S1 – chr12). These interactions are reminiscent of the mating type loci of *Saccharomyces cerevisiae* [141,144], however, examination of the underlying sequence at these loci did not reveal candidate genetic elements that would explain such strong interactions. The second identifiable element was the clustering of telomeres within and between the termini of chromosomes (see green circles in Fig. 2a). The separate clustering of centromeres and telomeres, typically at opposed ends of the nucleus, corresponds to the description of Rabl-like genome organisation, commonly seen in the genomes of some plants, as well as yeast with similarly small genomes [145].

Chromatin conformation capture methods like Hi-C are an invaluable tool for the investigation of 3D genomic architecture as applied here, enabling final scaffolding of genome assembly, identification of chromosomal rearrangements and features such as centromeres, telomeres, and loop anchors. Chromosomal rearrangements, fusions and translocations as seen here between chr04 and chr06 and between chr09 and chr010 can occur naturally or otherwise in animals, fungi and plants [146,150]. However, the formation of dicentric chromosomes, for example, are known to be unstable and typically result in the deletion or inactivation of one of centromeres when fixed in a population (indicated by a ‘?’ in Fig. 2c) [[151,152]]. Large structural genome rearrangements have however been previously observed in the PPEs *P. celeri* [153] and *O. tauri* [23], and can be important factors in population adaptation and divergence [154,155] and genome evolution and diversity [147,150]. Additionally, centromere characterization has become invaluable in molecular and synthetic biology and has enabled the development of yeast plasmid vectors [156,157], artificial chromosomes, neochromosomes [158,160] and episomes [161,163].

### Selenocysteine proteins and metabolism

Genomic selenoprotein composition has been associated with algal environmental, habitat and lifestyle adaptation [164]. Thus, we examined the selenoprotein composition of *Picochlorum* SENEW3. blastP matching of the *P. SENEW3* proteome against the algal selenoprotein database [164] identified 11 selenoproteins (10 families) out of the 25 selenoprotein families found in algae. This includes an additional six selenoproteins, aside from the five that have recently been identified in *P. SENEW3* [164]. In addition, the presence of the selenocysteine tRNA gene, tRNA-Sec(TCA), required for selenoprotein translation [165] was also identified. The best blastP matches for *P. SENEW3* selenoproteins against the algal selenoprotein database [164] included two glutathione peroxidases (GPXs) as well as possible selenoproteins K (SELENOK), T (SELENOT), and U (SELENOU). With additional matches to Thioredoxin reductase (TXNRD), Iron-sulphur oxidoreductase (FeSoxR), DNA-[protein]-cysteine methyltransferase selenoprotein (MDP), Methionine sulfoxide reductase A (MSRA) and Selenoprotein O (SELENOO) (Tables S15 and S16).

Selenium (Se) is an essential trace element in many organisms, it is co-translationally incorporated into the 21st amino acid selenocysteine (codon UGA) [165,167] and undertakes vital roles in both redox homeostasis and numerous cellular processes [168,171]. Despite identification of an additional six putative selenoproteins here, the Trebouxiophyceae, including *P. SENEW3*, appear to contain far fewer [164]. Among the aquatic green algae, the order Mamiellales, for example, has been found to possess the greatest number of selenoproteins (generally >20). In fact, most algal groups high in selenoproteins, like the Mamiellales, appear to live in marine environments. Whereas algae and plants inhabiting non-marine environments (i.e. freshwater rand terrestrial) have small selenoproteomes, often replaced with cysteine or expunged altogether [164,172, 173]. We speculate that the ancestor of *P. SENEW3*, along with others in the genus, initially adapted to freshwater, undergoing a reduction in selenoprotein content prior to re-adapting to marine and saline lagoon environments. This seems likely considering the closely related genus *Chlorella* predominantly inhabits freshwater habitats [174].

### Transporters

Transporter analysis using TransAAP [104], KEGG [107] and eggnog annotations [108,109] revealed a high number of likely transporters (416) largely similar to those predicted by Foflonker [26] [26] (Tables S17 and S18). Some of the most notable were the 39 annotated metal-ion transporters, with the most numerous being of the zinc (Zn2+)-iron (Fe2+) permease (ZIP) family, the CorA metal ion transporter (MIT) family, the P-type ATPase (P-ATPase) superfamily and the ATP-binding Cassette (ABC) superfamily for transport of zinc (Zn), magnesium (Mg), cobalt (Co) and Copper (Cu). Three iron transporters were also identified, including a high affinity iron permease 1, as well as transporters for the toxic heavy metals cadmium/zinc (Cd/Zn), chromate (CrO^2−^), nickel (Ni), tellurium (Te), metal tolerance proteins A2 and C4 (MTPA2 and MTPC4) and arsenite (As III), though this may in fact be involved in chlorophyll biogenesis in algae [175].

Metal-ion transporters form vital components in the interactions of algae with their environment, and function in the scavenging and transport of essential cofactors and in initial response to fluctuations in extracellular metal concentrations. The abundance of these metal transporters likely represents their requirement in the ‘light’ and ‘dark’ reactions of photosynthesis [176,177]. In addition, the presence of heavy metal transporters could represent potential avenues for metal tolerance in *P. SENEW*, which should be investigated further. Indeed, many microalgae are being investigated for potential heavy metal phytoremediation applications [178,182]. A recent review by Goswami *et al*. [183], investigating phytoremediation of wastewater by *Picochlorum* strains suggest a high level of strain variability in tolerance to heavy metal contaminated water.

We identified numerous sodium ion (Na^+^): proton (H^+^)/phosphate (PO₄³⁻) symporters and antiporters, potassium/sodium ion antiporters and two inward rectifier potassium channel transporters. An additional four (total of seven) mechanosensitive ion channel proteins, and nine calcium ion (Ca^2+^) transporters were also identified. The sodium, potassium, and calcium transporters in *P. SENEW3* likely contribute to its broad salinity tolerance [26,28]. This euryhaline characteristic of *P. SENEW3* is most likely expanded by the additional mechanosensitive ion channels, which are responsible for transducing physical force into electrochemical signals. In particular, mechanosensitive channels of small conductance (MscS) are responsible for management of cytosolic osmotic pressure and can act as emergency release valves by rapidly jettisoning cellular osmolytes under hypoosmotic shock, preventing cell lysis [184,188].

The final set of prominent transporters identified were five putative bestrophin proteins, a family of calcium (Ca^2+^) activated chloride (Cl^-^) channels that mediate the transport of monovalent anions (largely Cl^-^) in response to intracellular Ca^2+^ [189,190]. Aside from transporting Cl^-^, bestrophin channels have also been found to be highly permeable to bicarbonate (HCO_3_-) [191]. HCO_3_- is utilized as a form of inorganic carbon in algal CO_2_-concentrating mechanisms (CCMs) to increase the CO_2_:O_2_ ratio at the active site of ribulose-1,5-bisphosphate carboxylase-oxygenase (RuBisCO). This enhances the efficiency of photosynthetic carbon fixation in water where CO_2_ diffusion is limited [192,193]. For example, in the green alga *Chlamydomonas reinhardtii* bestrophin channel proteins have been found to be upregulated under low CO_2_ conditions [193,194], and have been proposed as an essential component of CCM HCO_3_-delivery to the thylakoid lumen from the chloroplast stroma [195]. It is therefore reasonable to speculate that bestrophin channel transporters play a key role in algal CCMs and have therefore been retained despite genome minimization and streamlining present in *P. SENEW3* and other picoeukaryotic phytoplankton [9,23].

### CAM and C4 photosynthetic pathways

**Fig. 5.**
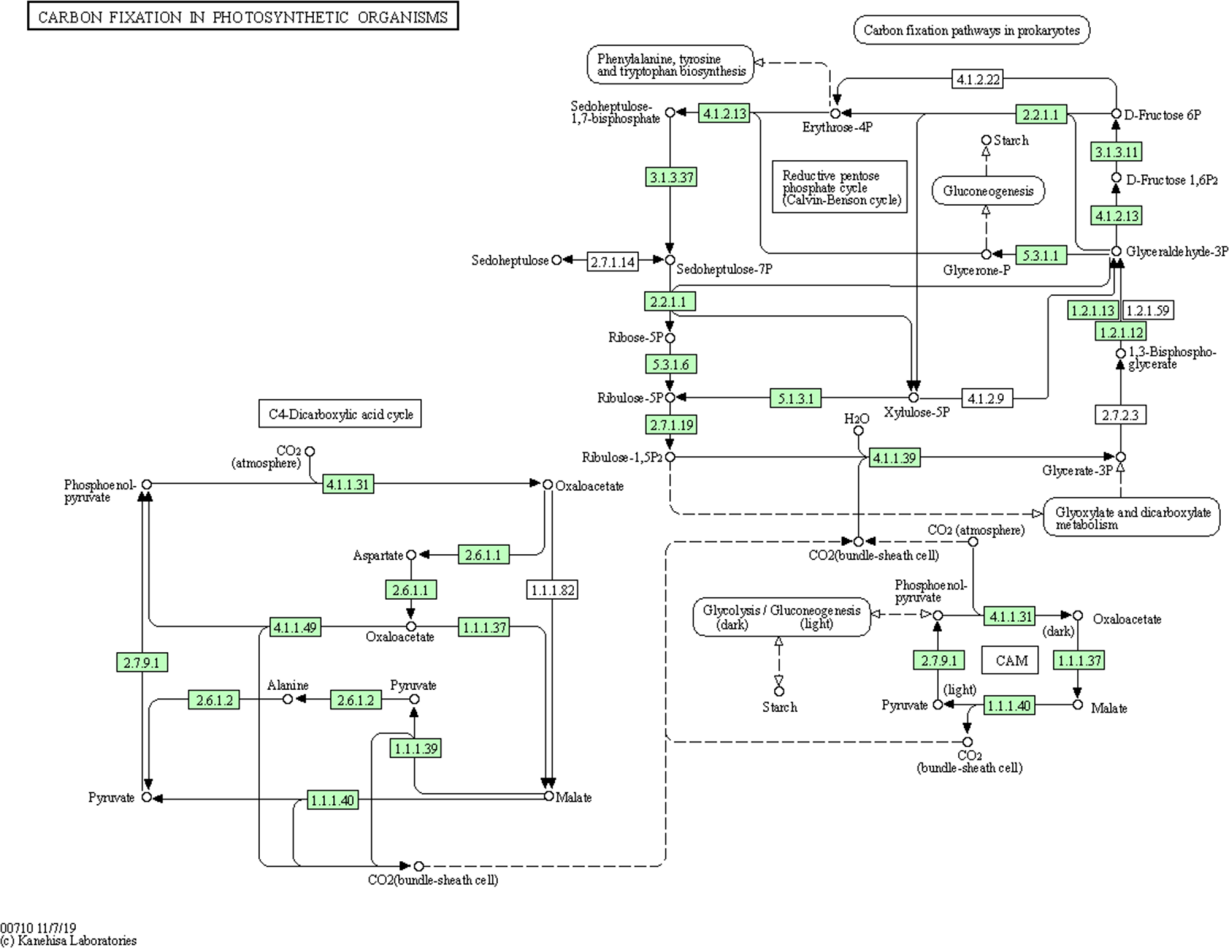
Kyoto Encyclopaedia of Genes and Genomes (KEGG) map of carbon fixation in photosynthetic organisms made with KEGG Mapper [209,210]. Enzymes present in the *P. SENEW3* genome are highlighted in green, identifying genes necessary for both crassulacean acid metabolism (CAM) and C_4_ (Hatch-Slack / C_4_-Dicarboxylic acid cycle) photosynthesis.

Photosynthesis by phytoplankton and algae in the world’s aquatic habitats accounts for a large proportion of global primary production [196,197]. Analysis of carbon fixation related genes and biochemical pathways present in the *P. SENEW3* genome revealed the genetic capacity for both Crassulacean acid metabolism (CAM) and Hatch-Slack (C_4_) photosynthesis (Table S14). As displayed in Fig. 5, the *P. SENEW3* genome encodes the required enzymes for both CAM photosynthesis and NAD-malic enzyme (NAD-ME) type, and phosphoenolpyruvate carboxykinase (PCK) type C_4_ photosynthesis. Most notably this included single copies of genes encoding for the three core C_4_ enzymes phosphoenolpyruvate carboxylase (PEPCase, EC: 4.1.1.31), phosphoenolpyruvate carboxykinase (PEPCKase EC: 4.1.1.49) and pyruvate phosphate dikinase (PPDKase, EC: 2.7.9.1) [198,199].

C_4_ photosynthesis concentrates CO_2_ for fixation by RuBisCO and is initiated by the carboxylation of phosphoenolpyruvate (PEP) by PEPCase to form a four-carbon acid compound oxaloacetate (OAA) [198,199]. CO_2_ incorporated by this mechanism, lowers photorespiration and water use, improving photosynthetic efficiency under low CO_2_, dry and/or high temperature conditions [198,199]. While CAM is highly prevalent in algae, C_4_ photosynthesis is relatively infrequent [194]. For example, in the green chlorophyte algae C_4_-like photosynthesis has only been identified in a handful of lineages including the microalgae *Ostreococcus tauri*, *Micromonas* sp. CCMP1545 and *Micromonas* sp. RCC299 [9,23], as well as the macroalgae *Udotea flabellum* [200], *Ulva prolifera* [201,202] and *Ulva linza* [203,204]. It has been postulated that CAM and C_4_ photosynthesis likely arose in aquatic eukaryotes in response to the restricted availability of dissolved CO_2_ in water, thereby maximising carbon acquisition and enhancing growth rates [205,207]. In *Ulva prolifera* for example, CO_2_ is preferentially assimilated by the C_3_ pathway under normal conditions but assimilated via a HCO_3_^-^ CAM mechanism under low CO_2_ conditions and via a C_4_ pathway under high-light irradiance [201,202]. Further experimental work will be required to demonstrate the activity and localization of the identified genes in CAM and C_4_ photosynthesis. Nevertheless, we think it likely that the genetic capacity for CAM and C_4_ photosynthesis in *P. SENEW3* could have been acquired as a response to its highly variable aquatic environment.

## Conclusion

New techniques and methodologies now enable improved characterization of genomes, their features, and their structures. In this study we re-investigated the genome of the PPE alga *P. SENEW3* utilizing short-read, long-read, and Hi-C chromosome conformation capture sequencing, resulting in a thoroughly annotated full chromosomal assembly. The *P. SENEW3* genome contained a low repetitive sequence content and a minimal complement of genes, with few duplications. Hi-C contact mapping revealed the *P. SENEW3* genome to have a Rabl-like structure and enabled identification of putative centromeres and a candidate CenH3 variant. These results form the basis for further centromere characterisation and possible development of native plasmids and artificial chromosomes. Despite detection of additional selenoproteins, overall selenoprotein content remained low, especially in comparison to the picoeukaryotic Mamiellophyceae algae. We speculate this could be explained by adaptation from freshwater to saline environments during their evolutionary history. Finally, alongside the C_3_ photosynthetic pathway, we identified the necessary genes for both CAM and NAD-ME/PCK type C_4_ photosynthesis. C_4_ photosynthesis is still uncommon in chlorophyte algae, the genetic capability for it may partially explain the unique robustness of this organism. This new fully annotated chromosomal assembly provides an improved resource for the molecular study of this unique organism and offers further insight into its unique environmental adaptations.

## supplementary material

10.1099/mgen.0.001223 Uncited Supplementary Material 1.

10.1099/mgen.0.001223 Uncited Table S1.

## Abbreviations

CAM: crassulacean acid metabolism
CCM: CO2-concentrating mechanism
CDS: protein coding sequence
CTAB: cetyltrimethyl ammonium bromide
EDTA: ethylenediaminetetraacetic acid
ONT: Oxford Nanopore Technologies
PFGE: pulsed field gel electrophoresis
PPE: photosynthetic picoeukaryote
*P. SENEW3*: *Picochlorum* sp. SENEW3
RuBisCO: Ribulose-1,5-bisphosphate carboxylaseoxygenase
TBE: Tris/Borate/EDTA
TE: transposable elements
