# Supplementary material for: Chromosome-scale assembly of the streamlined picoeukaryote Picochlorum sp. SENEW3 genome reveals Rabl-like chromatin structure and potential for C4 photosynthesis
Source: Microb Genom. 2024 Apr 16;10(4):001223. doi: 10.1099/mgen.0.001223 (PMC11092101; doi:10.1099/mgen.0.001223)
Supplement: Uncited Supplementary Material 1. [file mgen-10-01223-s001.pdf]

Supplementary

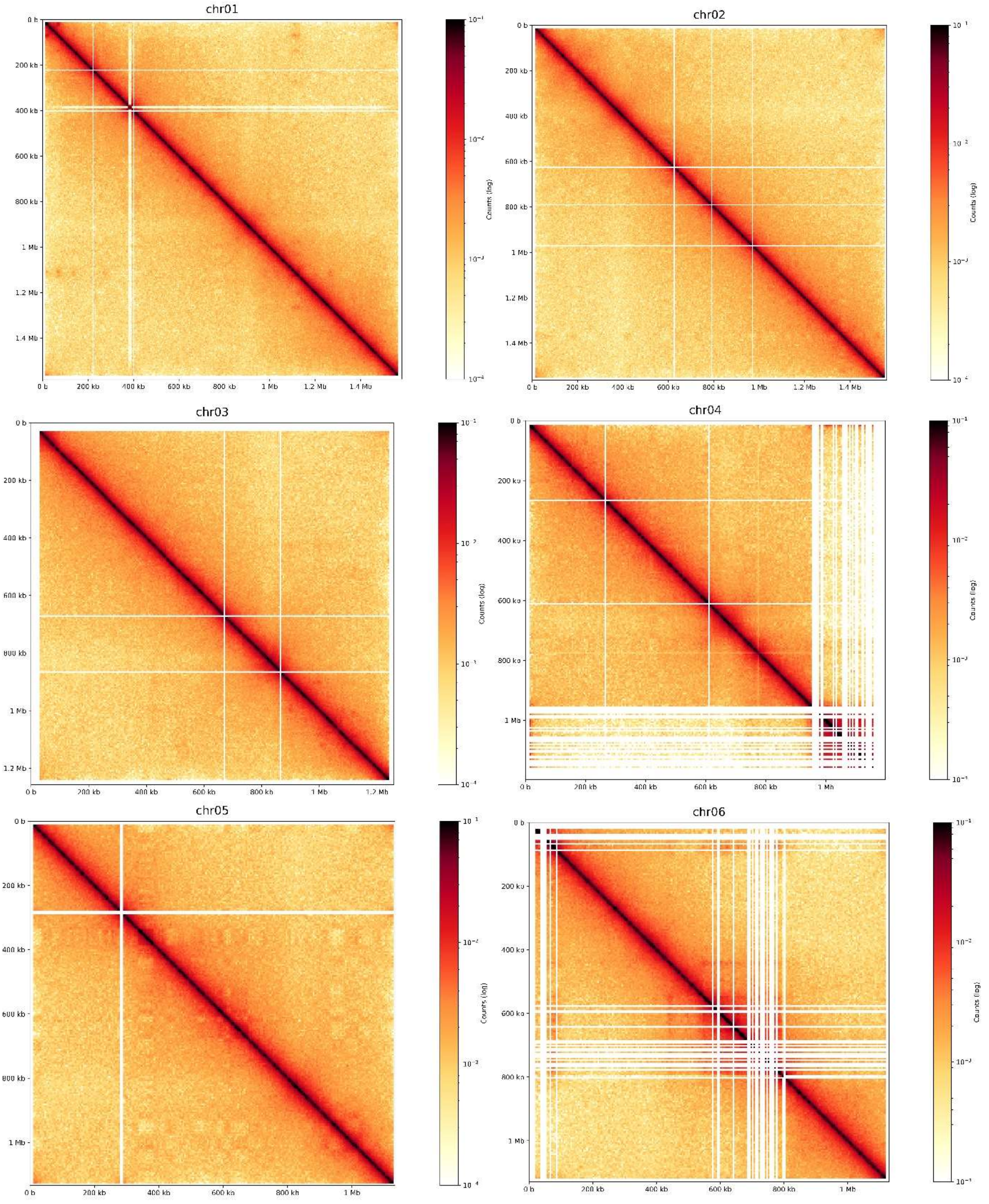

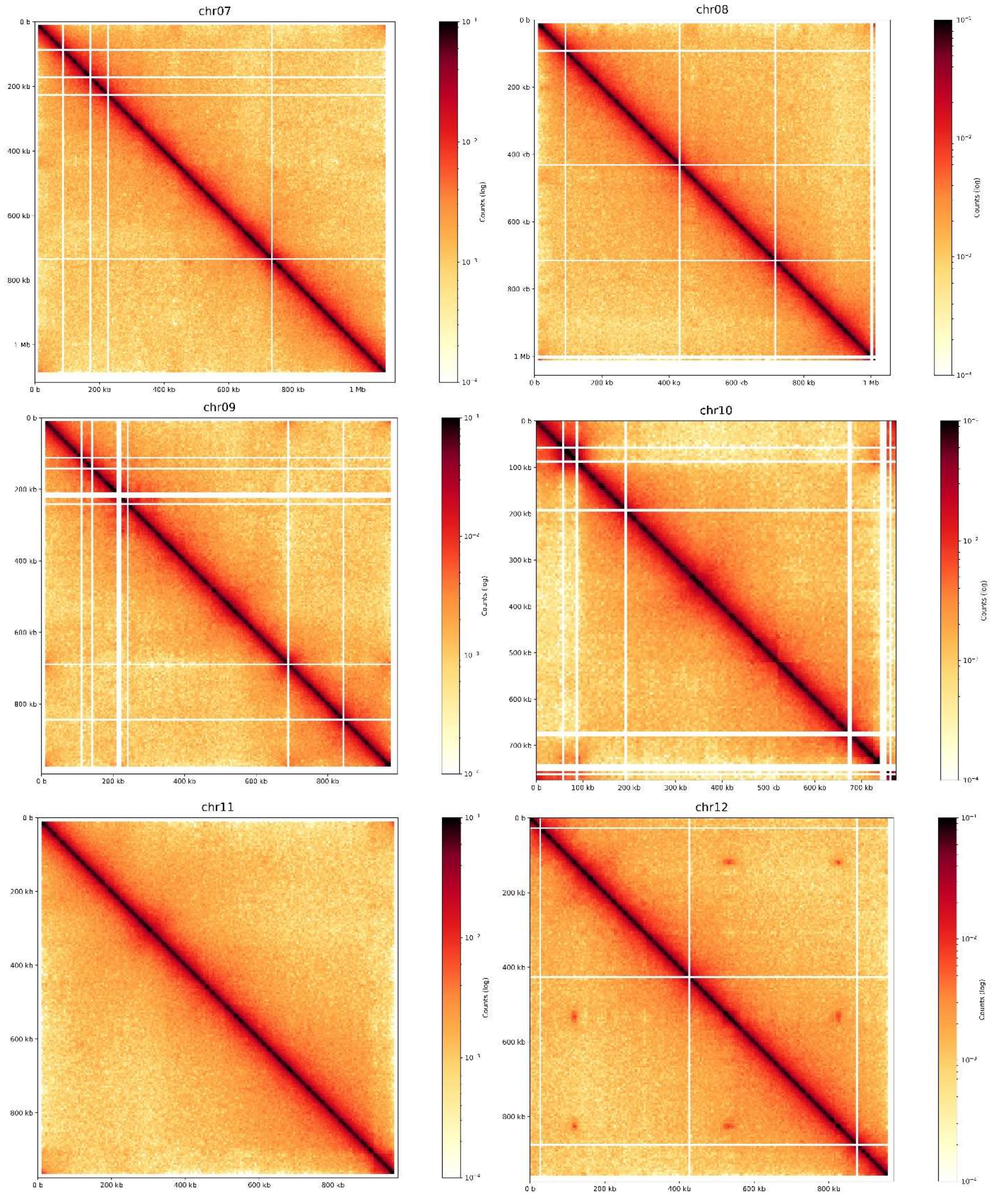

Supplementary Figure 1. Hi-C contact heat maps (balanced) at 5 Kbp resolution for nuclear genome primary assembly chromosomes (chr) 1 – 12. Scale (log) shows interaction frequency count per 5 Kbp bin.

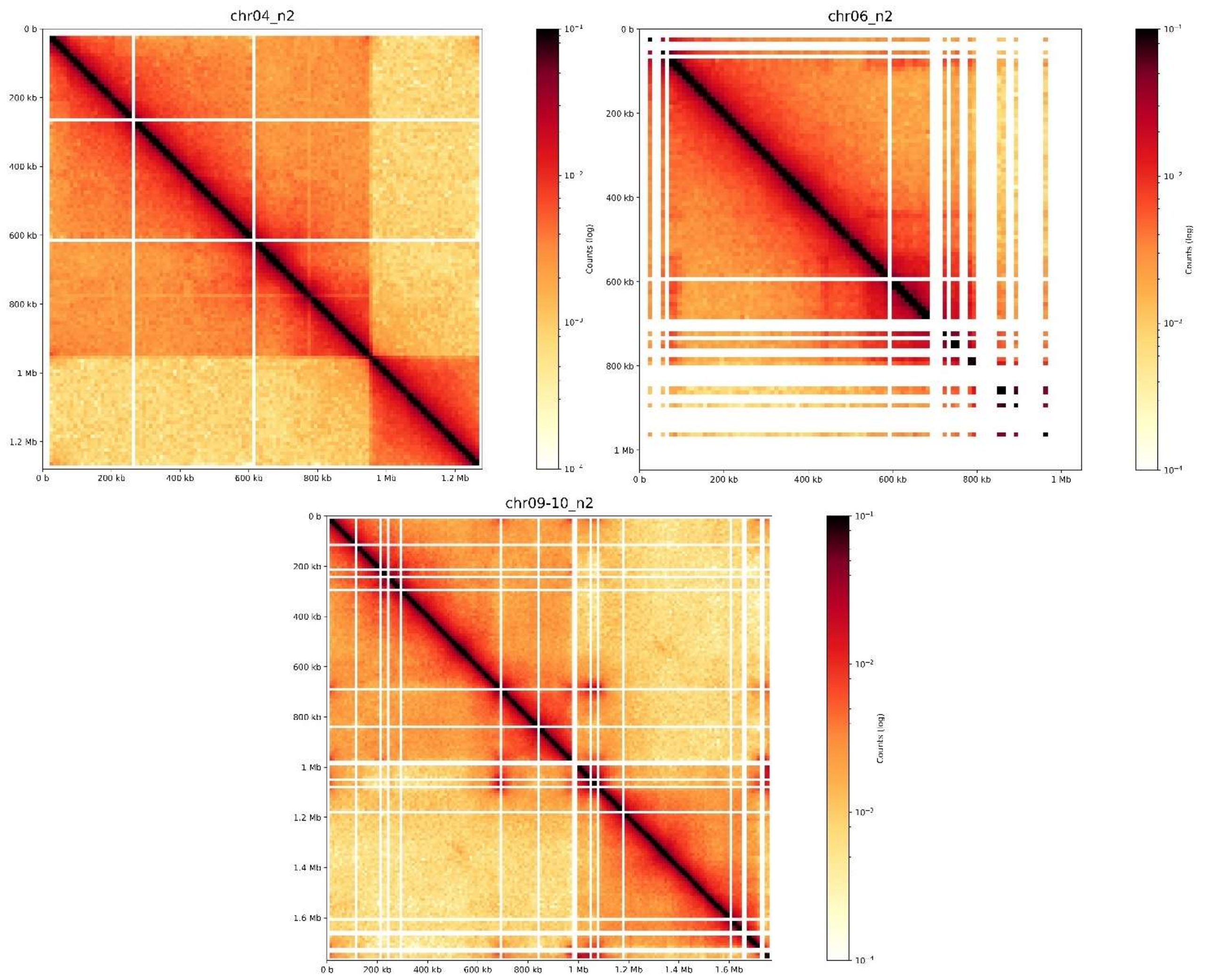

Supplementary Figure 2. Hi-C contact heat maps (balanced) at 5 Kbp resolution for nuclear genome alternative "n2" haplotype variant chromosomes (chr) chr04_n2, chr06_n2 and chr09-10_n2). Scale (log) shows interaction frequency count per 5 Kbp bin.

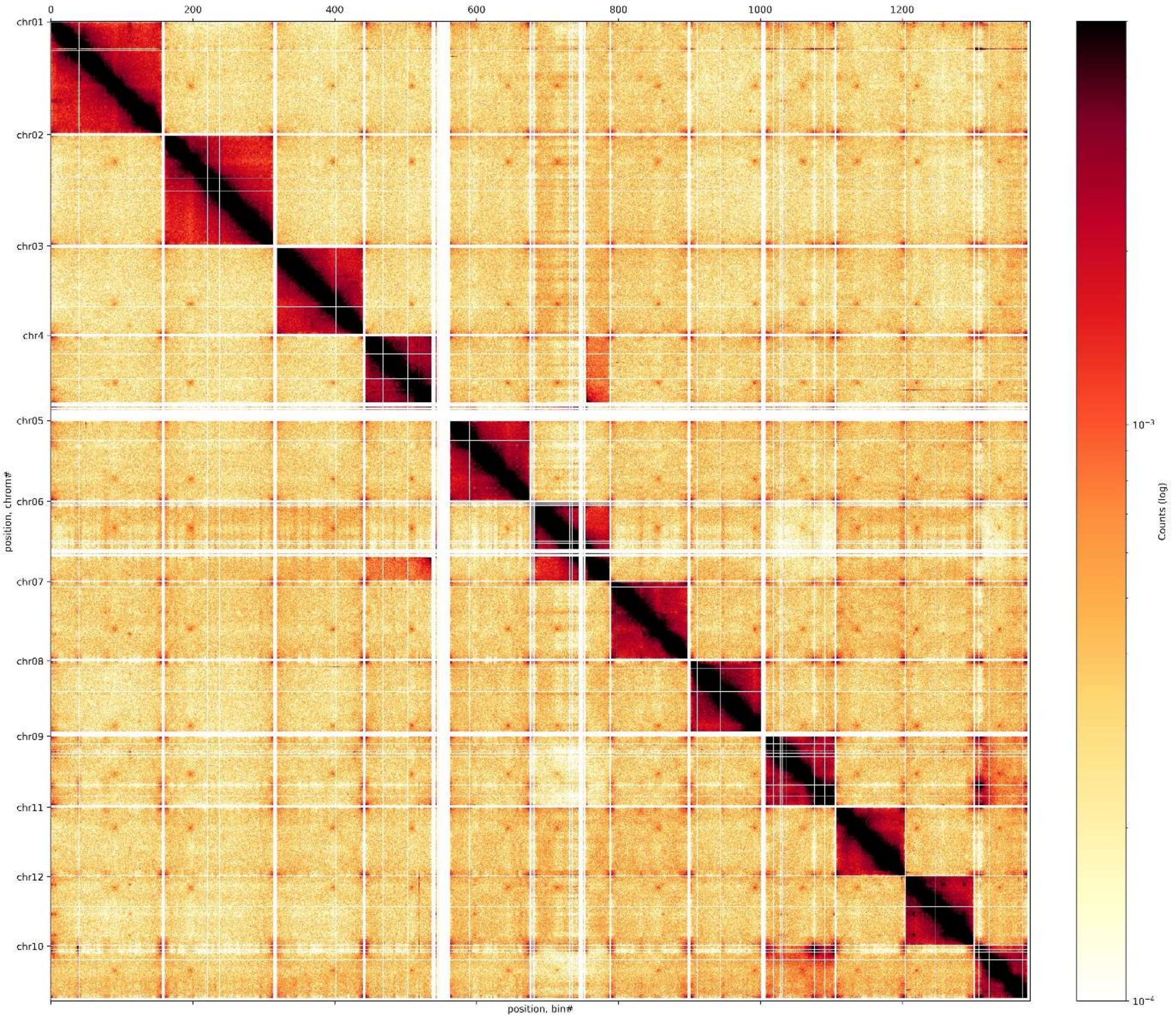

Supplementary Figure 3. Hi-C contact heat maps (balanced) of final full primary nuclear genome (chromosomes 1 – 12) at 10 Kbp resolution. Scale (log) shows interaction frequency count per 10 Kbp bin.

Supplementary Table 1. CHEF Mapper XA PFGE system run settings for separation of *Picochlorum* sp. SENEW3 chromosomal DNA.

**Setting**	**Stage / Slice 1**	**Stage / Slice 2**	**Stage / Slice 3**	**Stage / Slice 4**
Buffer	0.5 X TBE	0.5 X TBE	0.5 X TBE	0.5 X TBE
Temperature	12 °C	12 °C	12 °C	12 °C
Voltage	6	6	6	6
Calibration factor	1	1	1	1
Included angle	120°	120°	120°	120°
Switch time (linear ramped)	60 – 120 s	122.5 – 130 s	132.5 – 140 s	142.5 – 150 s
Run time	24 h	28 h	32 h	36 h

Supplementary Tables 2 - 22

Additional supplementary data tables are available in the supplementary data spreadsheet.
